# Supplementary material for: SK119, a Novel Shikonin Derivative, Leads to Apoptosis in Melanoma Cell Lines and Exhibits Synergistic Effects with Vemurafenib and Cobimetinib
Source: Int J Mol Sci. 2022 May 19;23(10):5684. doi: 10.3390/ijms23105684 (PMC9145845; doi:10.3390/ijms23105684)
Supplement: Supplementary file 1 [file ijms-23-05684-s001.zip › ijms-1726895-SI.pdf]

SUPPLEMENTAL MATERIAL TO:

SK119, a novel shikonin derivative, leads to apoptosis in melanoma cell lines and exhibits synergistic effects with vemurafenib and cobimetinib

Nadine Kretschmer [1,2], Christin Durchschein [1], Antje Hufner [3], Beate Rinner [2], Birgit Lohberger [4*], Rudolf Bauer [1]

[1] Institute of Pharmaceutical Sciences, Department of Pharmacognosy, University of Graz, Beethovenstr. 8, 8010 Graz, Austria; nadine.kretschmer@uni-graz.at; christin.durchschein@hotmail.com; rudolf.bauer@uni-graz.at

[2] Division of Biomedical Research, Medical University Graz, Roseggerweg 48, 8036 Graz, Austria; beate.rinner@medunigraz.at

[3] Institute of Pharmaceutical Sciences, Department of Pharmaceutical Chemistry, University of Graz, Universitaetsplatz 1, 8010 Graz, Austria; antje.huefner@uni-graz.at

[4] Department of Orthopedics and Trauma, Medical University Graz, Auenbruggerplatz 5, 8036 Graz, Austria; birgit.lohberger@medunigraz.at

* Correspondence: birgit.lohberger@medunigraz.at

Content:

1. Additional NMR spectra of SK119 in $CDCl_3$

2. Additional NMR spectra of SK119 in benzene-d6

3. Structures of known dimers of shikonin

4. References

5. Uncropped files (western blots)

1. Additional NMR spectra of SK119 in $CDCl_3$

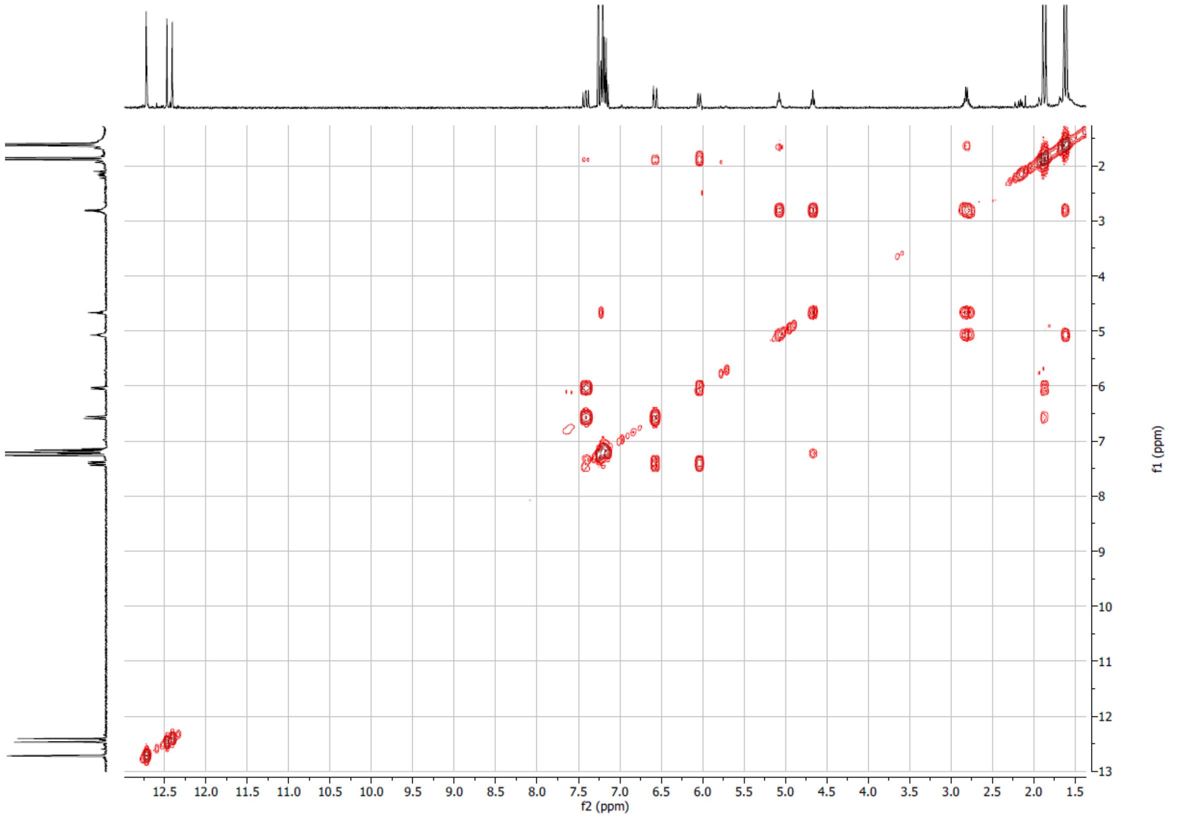

Figure S1. ^{1}H,^{1}H COSY spectrum ($CDCl_3$, 400 MHz) of SK119

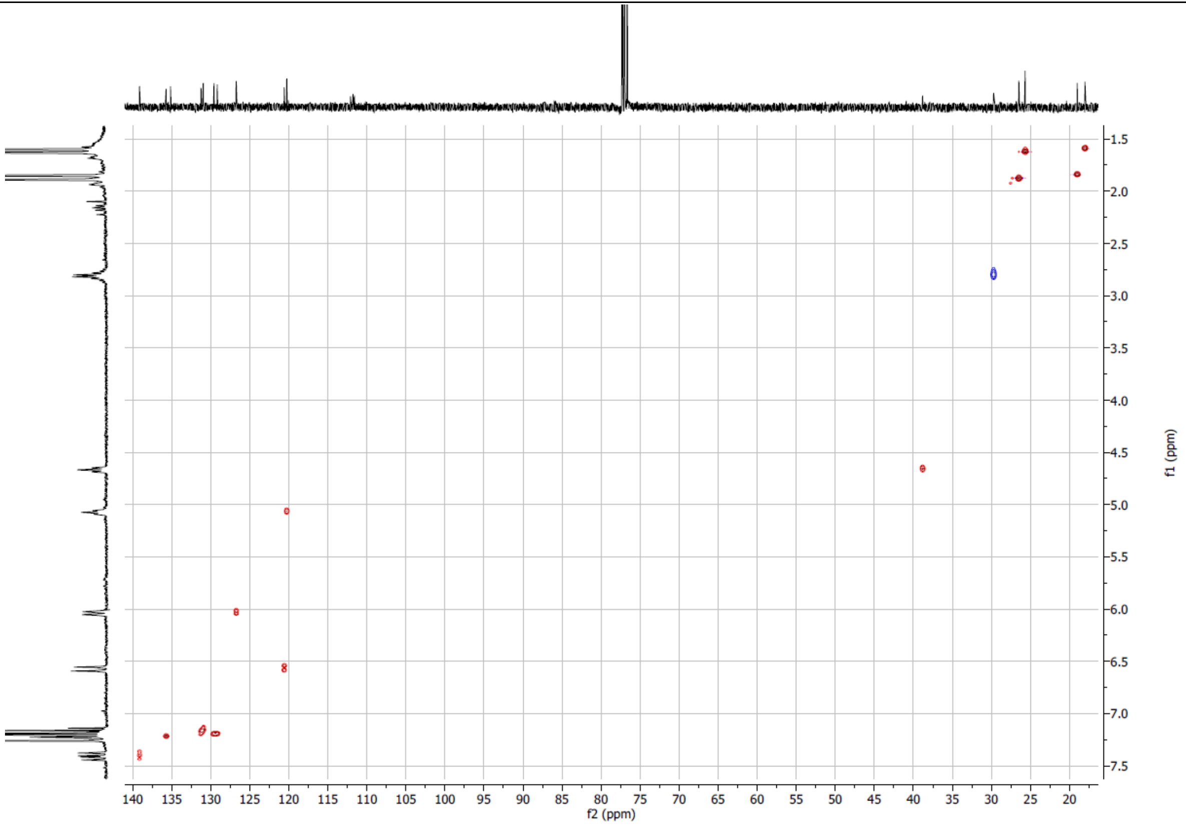

Figure S2. ^{1}H,^{13}C DEPT-HSQC spectrum ($CDCl_3$, 400 MHz) of SK119

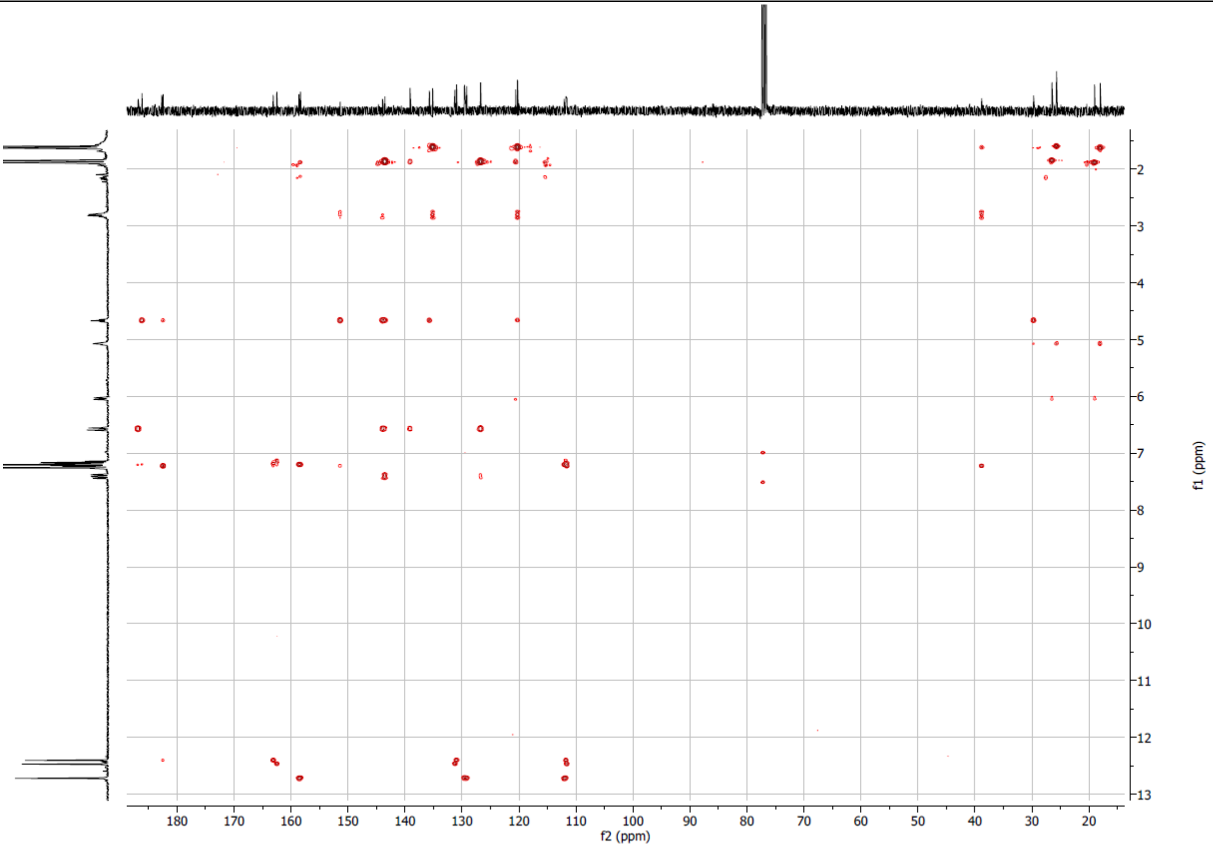

Figure S3. $^{1}H,^{13}C$ HMBC spectrum ($CDCl_3$, 400 MHz) of SK119

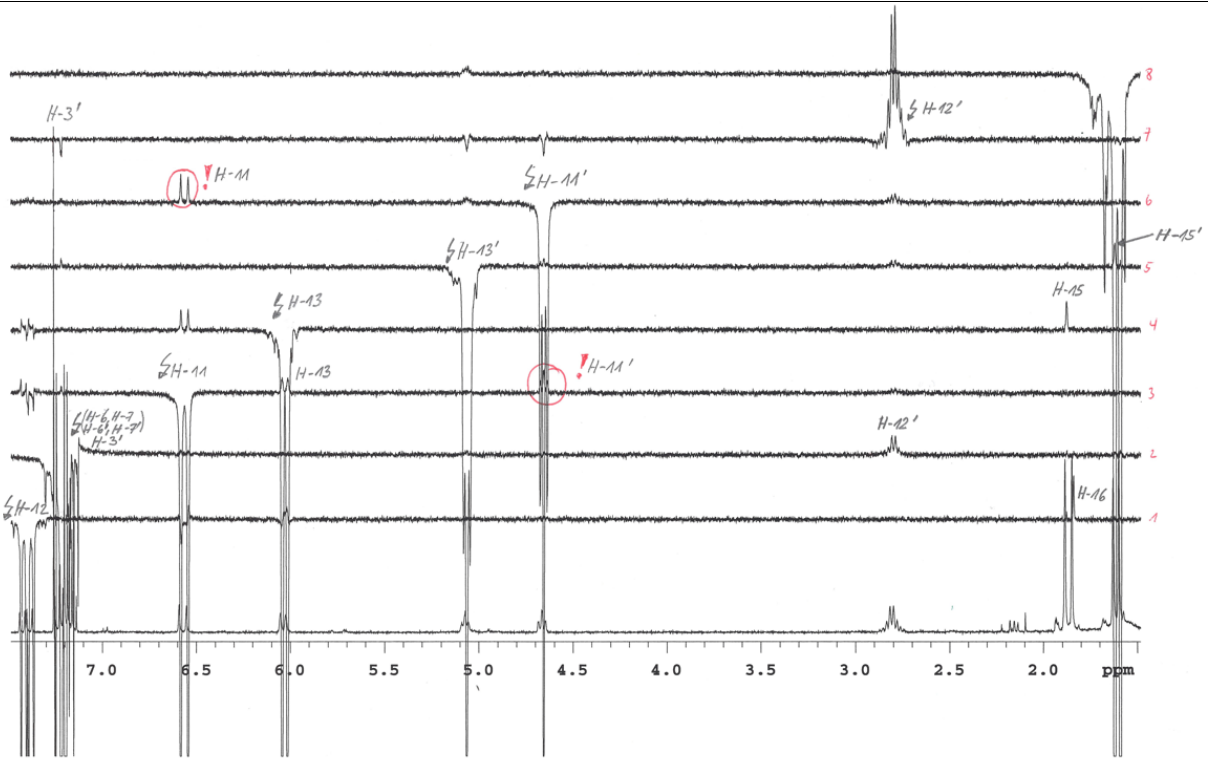

Figure S4. NOE spectra ($CDCl_3$, 400 MHz) of SK119

2. Additional NMR spectra of SK119 in C_6D_6

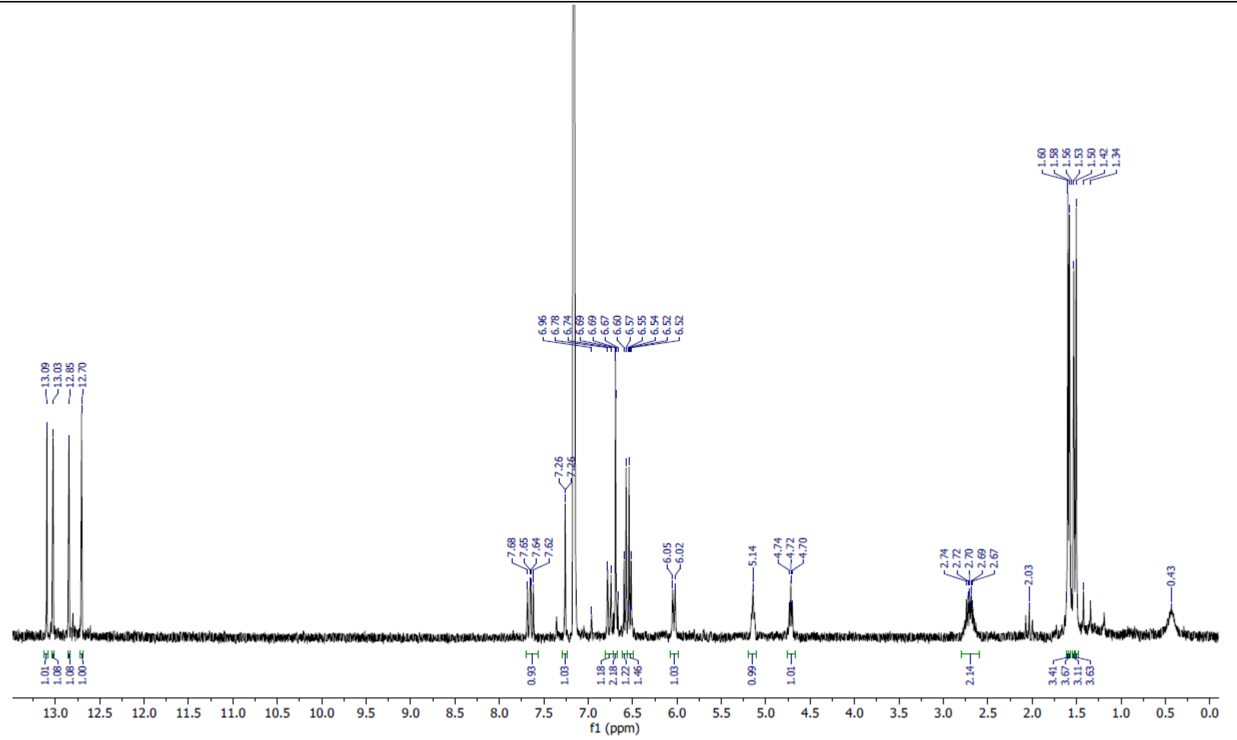

Figure S5. ^{1}H NMR spectrum (benzene-d6, 400 MHz) of SK119

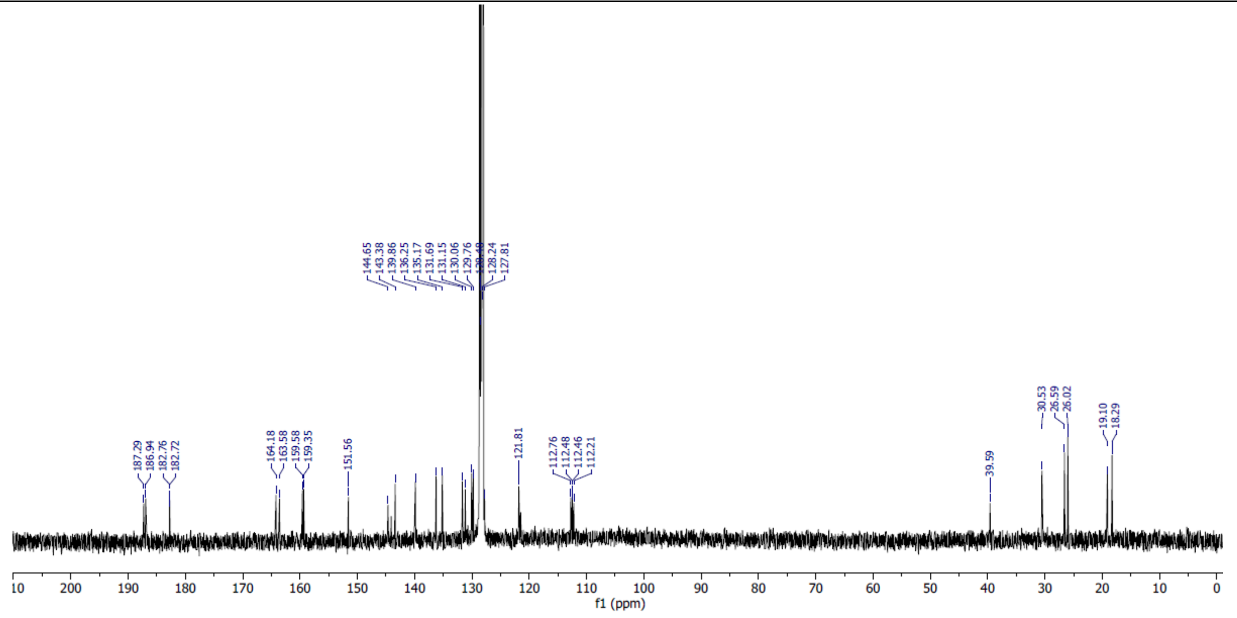

Figure S6. ^{13}C NMR spectrum (benzene-d6, 100 MHz) of SK119

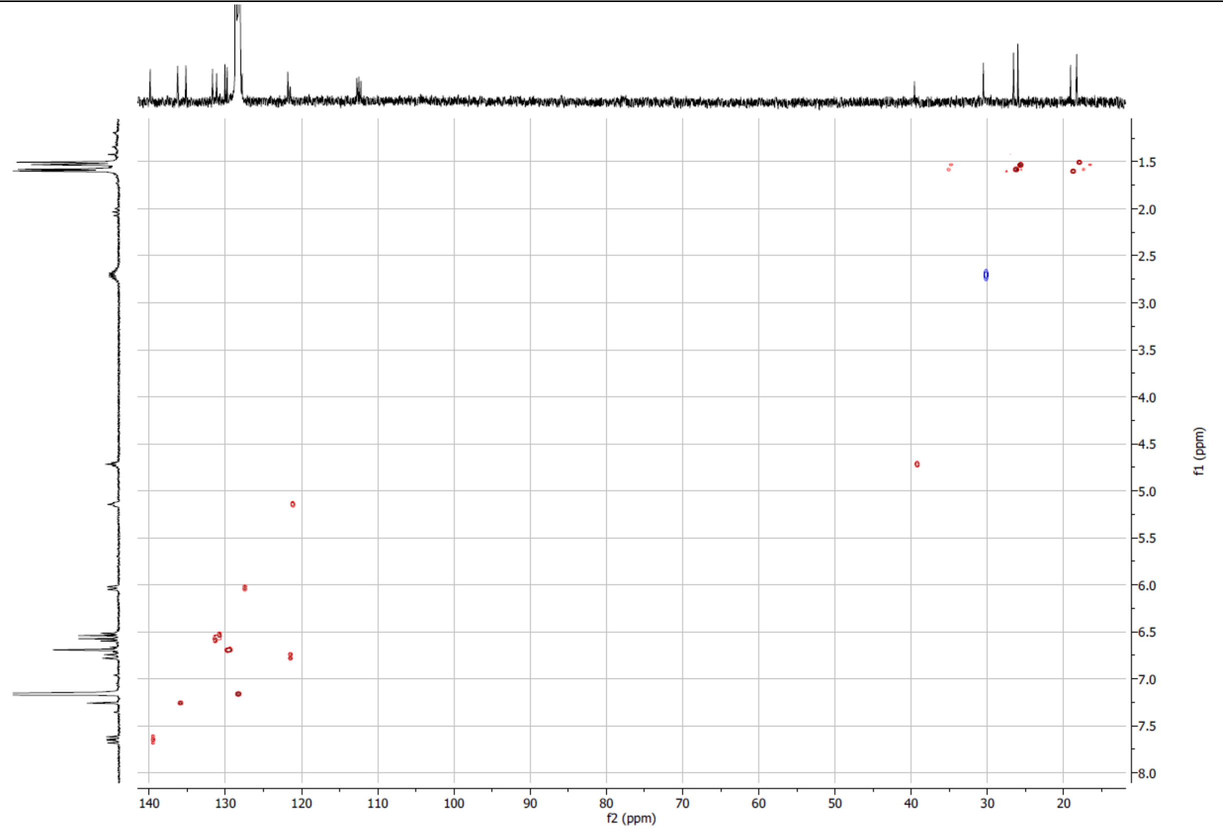

Figure S7. ^{1}H,^{13}C DEPT-HSQC spectrum (benzene-d6, 400 MHz) of SK119

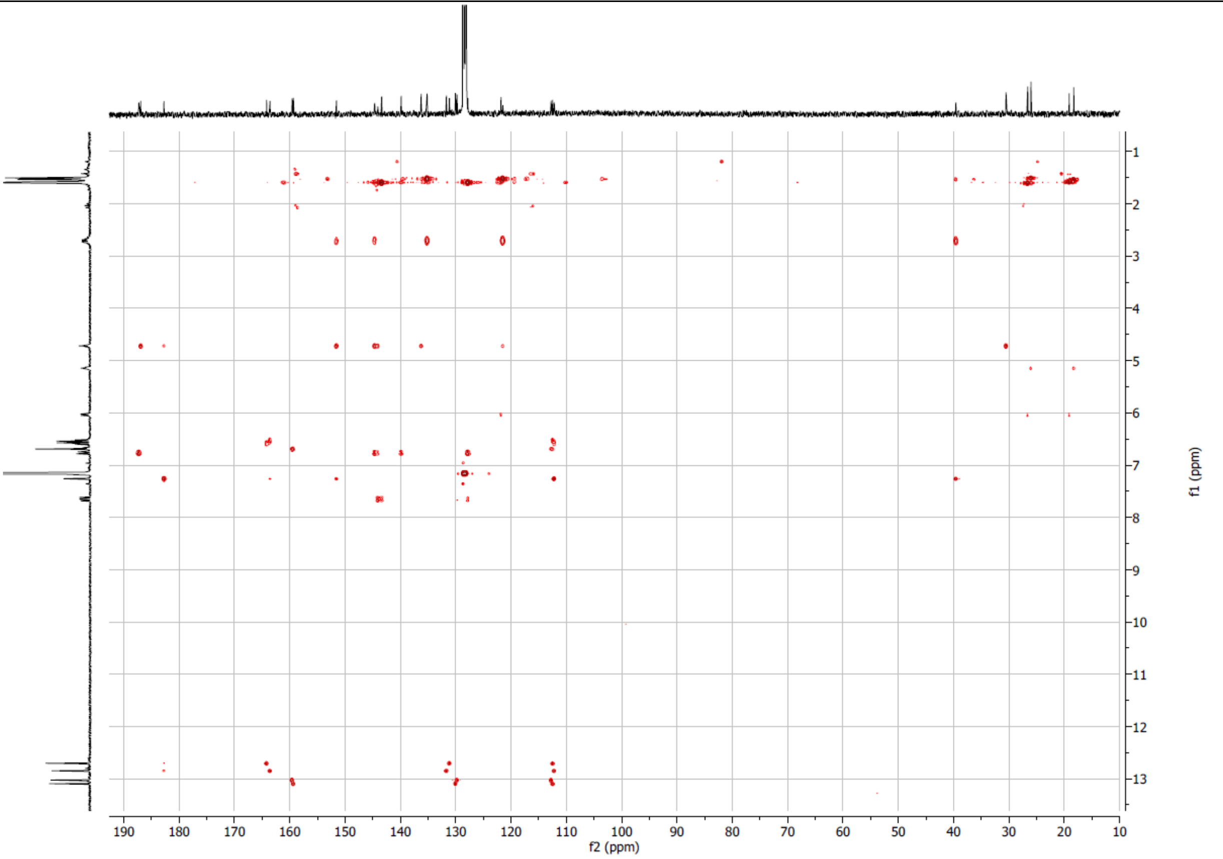

Figure S8. ^{1}H,^{13}C HMBC spectrum (benzene-d6, 400 MHz) of SK119

3. Structures of known dimers of shikonin

C,C bonds between two shikonin monomers can be established via the phenolic ring A (positions 6 or 7), the quinone ring B (position 3) or the side chain (position 11). In SK119, the side chain of one monomer is directly attached to ring B of the other monomer (C-11 to C-3 of shikonin). The only known compound with a similar connectivity is shikometabolin C, with connection of the shikonins between C-11 and C-3 and an additional direct connection between the two positions 3. Both side chains are directly bound in Shikometabolin D and arnebidin. Further condensations resulted in the hexa and hepta cyclic structures (Meselhy et al., 1994a; Meselhy et al., 1994b; Min et al., 2000; Ahmed et al., 2014).

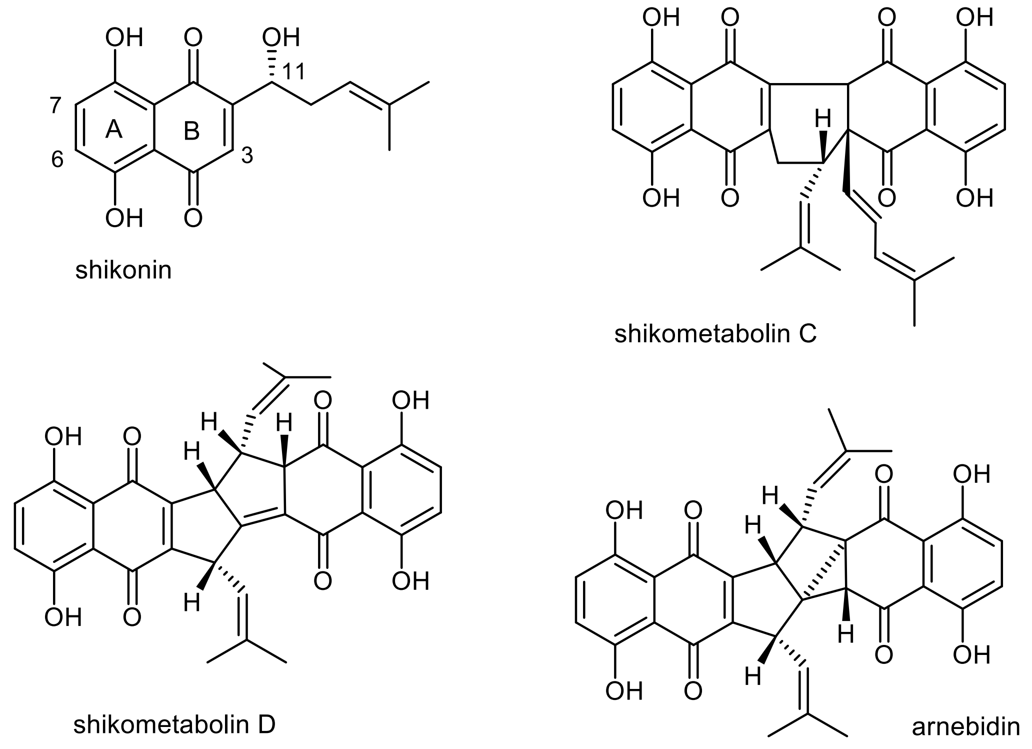

Figure S9. Shikonin and known dimers with side chain connection to quinone ring B

The side chain of one monomer is usually attached to ring A of the other monomer, either in position 6 or 7, as exemplified by vaforhizin, iso-vaforhizin and their acyl derivatives. Often further condensation leads to pentacyclic compounds like shikometabolines A and B, which may be acylated to acetates, valerates, isovalerates, β,β dimethylacrylates etc. or ethylated (Meselhy et al., 1994; Min et al., 2000; Spyros et al., 2005; Assimopoulou et al., 2008; Noula et al., 2010; Ali et al., 2011; Liao et al., 2015; Dong et al., 2017; Feng et al., 2020; Cao et al. 2020,

Liao et al., 2020). Yang et al. give the structures of three of these compounds but they mix up the naming of shikometabolines A, B and E (Yang et al. 2015).

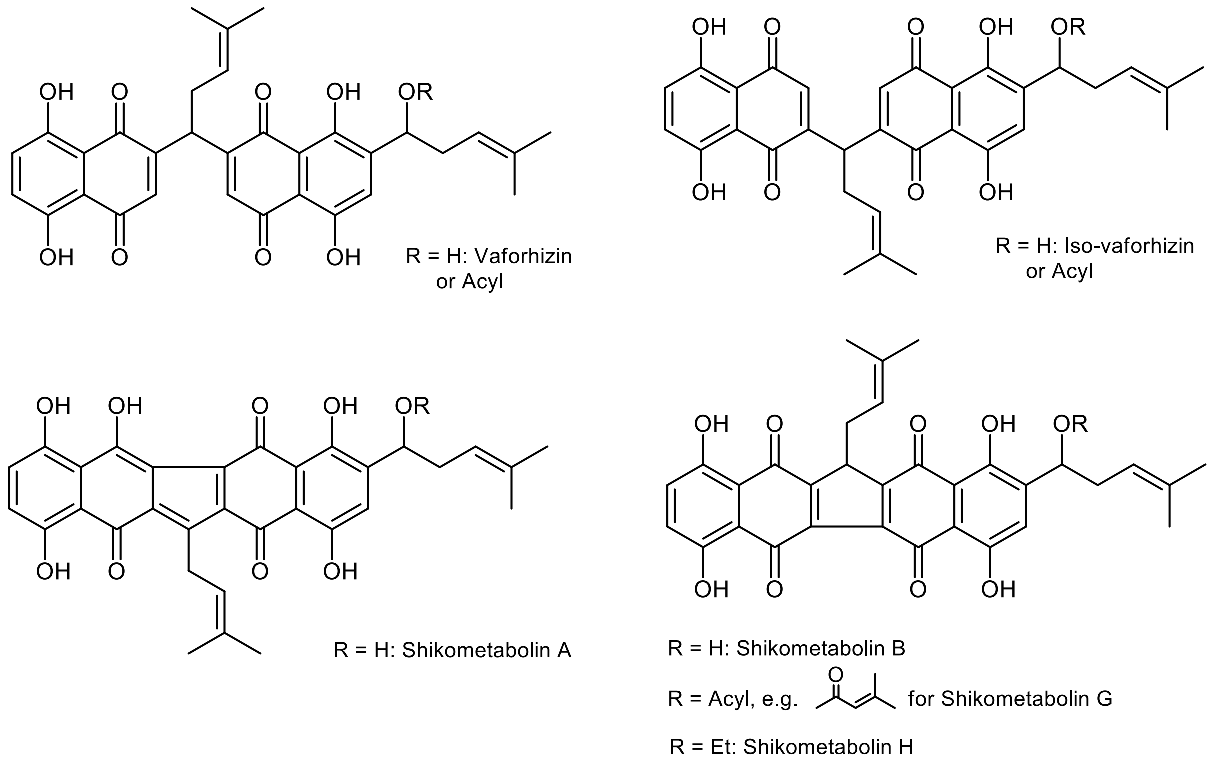

Figure S10. Shikonin and known dimers with side chain connection to phenol ring A

Dimerisations without participation of the side chains is rare, known products are presented in Figure S11 (Meselhy et al., 1994; Assimopoulou et al., 2008).

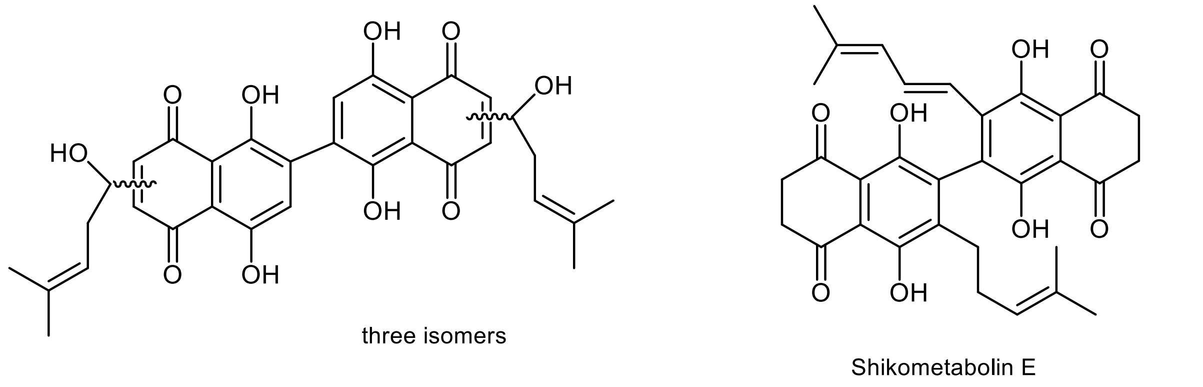

Figure S11. Shikonin and known dimers with direct connection of two rings A or two rings B

4. References

1. Ahmed, A.F.; Saad, H.-E.A.; El-Karim, E.M.A. Two Polycyclic Geranylhydroquinone-Derived Metabolites from Roots of Arnebia hispidissima (Lehm.) DC. Molecules **2014**, 19, 5940-5951. https://doi.org/10.3390/molecules19055940

2. Ali, A.; Assimopoulou, A.N.; Papageorgiou, V.P.; Kolodziej, H. Structure/Antileishmanial Activity Relationship Study of Naphthoquinones and Dependency of the Mode of Action on the Substitution Patterns *Planta Med*, 2011, *77*, 2003–2012. DOI: 10.1055/s-0031-1280092

3. Assimopoulou, A.N.; Ganzera, M.; Stuppner, H.; Papageorgiou, V.P. Simultaneous determination of monomeric and oligomeric alkannins and shikonins by high-performance liquid chromatography–diode array detection–mass spectrometry, *Biomed Chromatogr* **2008**, *22*, 173–190. DOI: 10.1002/bmc.912

4. Cao, H.; Zhang, W.; Liu, D.; Hou, M.; Liu, S.; He, W.; Lin, J.; Shao, M. Identification, in vitro evaluation and modeling studies of the constituents from the roots of Arnebia euchroma for antitumor activity and STAT3 inhibition. *Bioorg Chem* **2020**, *96*, 103655. DOI: 10.1016/j.bioorg.2020.103655

5. Dong, M.; Liu, D.; Li, Y.H.; Chen, X.Q.; Luo, K.; Zhang, Y.M.; Li, R.T. Naphthoquinones from *Onosma paniculata* with potential anti-inflammatory activity. *Planta Med* **2017**, *83(7)*, 631-635. DOI: 10.1055/s-0042-120545

6. Feng, J.; Yu, P.; Zhou, Q.; Tian, Z.; Sun, M.; Li, X.; Wang, X.; Jiang, H. An integrated data filtering and identification strategy for rapid profiling of chemical constituents, with *Arnebiae Radix* as an example *J Chrom A* **2020**, *1629*, 461496. DOI: 10.1016/j.chroma.2020.461496

7. Liao, M.; Li, A.; Chen, C.; Ouyang, H.; Zhang, Y.; Xu Y.; Feng, Y.; Jiang, H. Systematic identification of shikonins and shikonofurans in medicinal Zicao species using ultra-high performance liquid chromatography quadrupole time of flight tandem mass spectrometry combined with a data mining strategy *J Chrom A* **2015**, *1425*, 158–172. DOI: 10.1016/j.chroma.2015.11.028

8. Liao, M.; Yan, P.; Liu, X.; Du, Z.; Jia, S.; Aybek, R.; Li, A.; Kaisa, S.; Jiang, H. Spectrum-effect relationship for anti-tumor activity of shikonins and shikonofurans in medicinal Zicao by UHPLC-MS/MS and chemometric approaches *J Chrom B* **2020**, *1136*, 121924. DOI: 10.1016/j.jchromb.2019.121924

9. Meselhy, M.R.; Kadota, S.; Tsubono, K.; Hattori, M.; Nambaa, T. Biotransformation of Shikonin by Human Intestinal Bacteria, *Tetrahedron* **1994**, *50(10)*, 3081-3098. DOI: 10.1016/S0040-4020(01)81108-X

10. Meselhy, M.R.; Kadota, S.; Tsubono, K.; Kusai, A.; Hattori, M.; Nambaa, T. Shikometabolins A, B, C and D, Novel Dimeric Naphthoquinone Metabolites Obtained from Shikonin by Human Intestinal Bacteria, *Tetrahedron Lett* **1994**, *35(4)*, 583-586. DOI: 10.1016/S0040-4039(00)75844-8

11. Min, B.S.; Meselhy, M.R.; Hattori, M.; Kim, H.M.; Kim, Y.H. Cytotoxicity of shikonin metabolites with biotransformation of human intestinal bacteria. *J Microbiol Biotechnol* **2000**, *10(4)*, 514-517. no DOI

12. Noula, E.; Samanidou, V.F.; Assimopoulou, A.N.; Papageorgiou, V.P.; Papadoyannis, I.N. Solid-phase extraction for purification of alkannin/shikonin samples and isolation of monomeric and dimeric fractions *Anal Bioanal Chem* **2010**, *397*, 2221–2232. DOI: 10.1007/s00216-010-3717

13. Spyros, A; Assimopoulou, A.N.; Papageorgiou, V.P. Structure determination of oligomeric alkannin and shikonin derivatives, *Biomed Chromatogr* **2005**, *19*, 498–505. DOI: 10.1002/bmc.470

14. Yang Y; Zhao, D. Yuan, K.; Zhou, G.; Wang, Y.; Xiao, Y.; Wang, C.; Xu, J.; Yang, W. Two new dimeric naphthoquinones with neuraminidase inhibitory activity from Lithospermum erythrorhizon *Nat Prod Res* **2015**, *29(10)*, 908–913. DOI: 10.1080/14786419.2014.957695

5. Uncropped files (western blots)

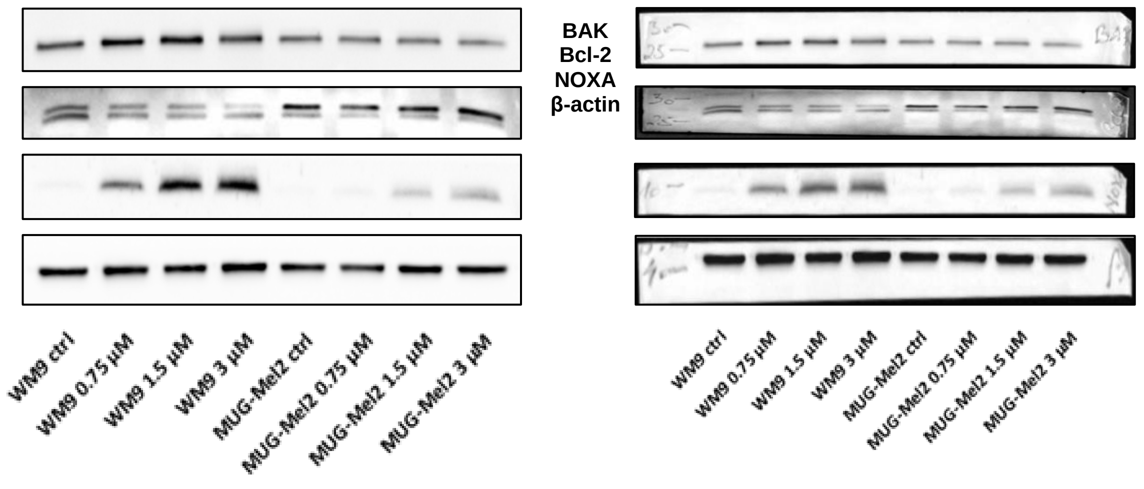

Figure S12. Uncropped files for all western blots of Figure 4B.

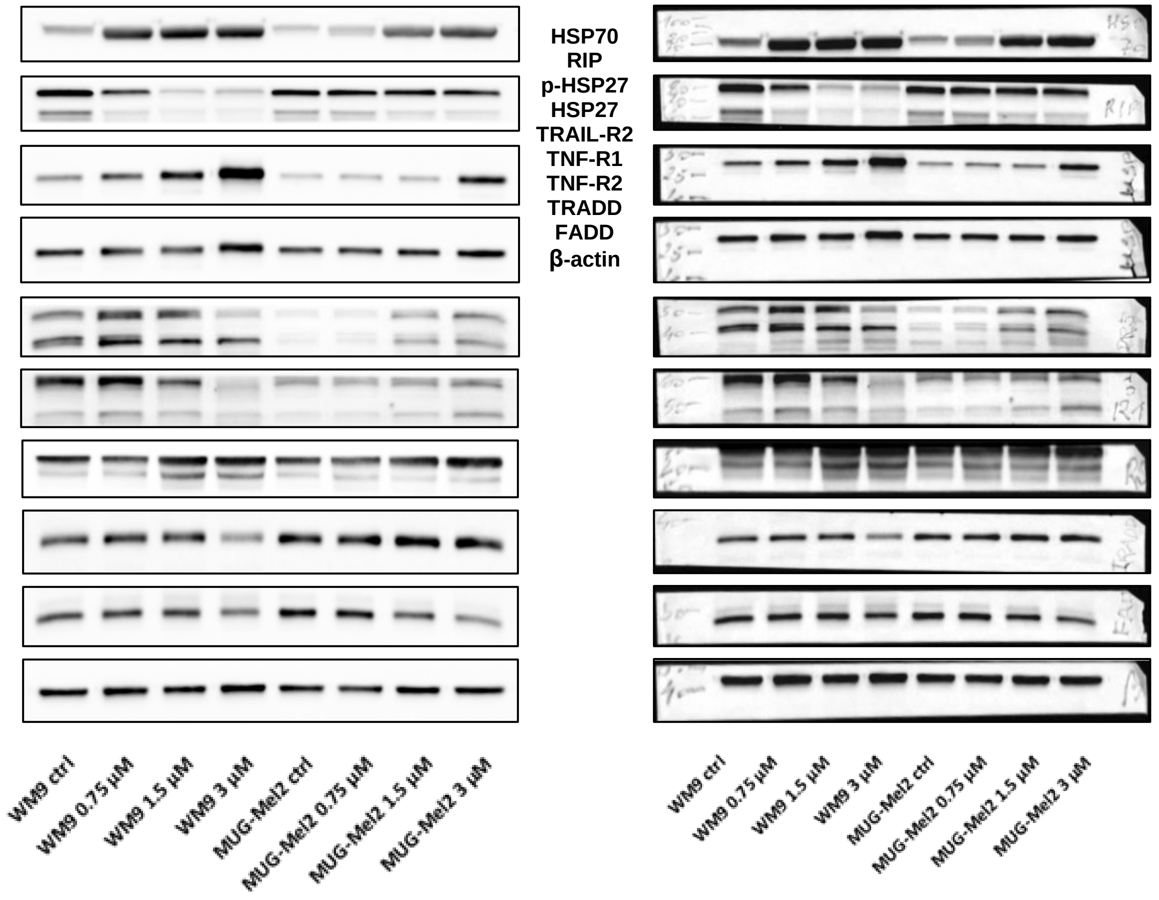

Figure S13. Uncropped files for all western blots of Figure 5A.
